# Role of a proteolysis-inducing factor (PIF) in cachexia induced by a human melanoma (G361)

**DOI:** 10.1038/sj.bjc.6690590

**Published:** 1999-08

**Authors:** P T Todorov, W N Field, M J Tisdale

**Affiliations:** Pharmaceutical Sciences Institute, Aston University, Birmingham , B4 7ET, UK

**Keywords:** cancer cachexia, proteolysis-inducing factor, G361 melanoma, sulphated glycoprotein

## Abstract

Human melanoma, G361, which induces cachexia in nude mice, has been shown to produce a proteolysis-inducing factor (PIF) of *M*_r_ 24 000, which is immunologically identical to that isolated from a cachexia-inducing murine tumour (MAC16). Biosynthetic labelling of G361 cells using a combination of [^35^S]sulphate and [6-^3^H]glucosamine gave a single component of *M*_r_ 24 000 after affinity chromatography employing a murine monoclonal antibody. The material contained both radiolabels and, after digestion with peptide *N*-glycosidase F, two fragments were produced of *M*_r_ 14 000 and 10 000 also containing both radiolabels. Digestion with *O*-glycosidase produced three fragments of *M*_r_ 14 000, 6000 and 4000, the first two of which contained both radiolabels, while the third only contained ^3^H. This digestion pattern is the same as that previously observed with PIF from the MAC16 tumour and is commensurate with one N-linked sulphated oligosaccharide chain of *M*_r_ 10 000, one O-linked sulphated oligosaccharide chain of *M*_r_ 6000 and a central polypeptide chain of *M*_r_ 4000 with some residual carbohydrate. When PIF from G361 cells was administered to female NMRI mice (20 g) a pronounced depression of body weight (1.36 ± 0.36 g; *P* < 0.0001 from control) was observed over a 24 h period without a decrease in either food or water consumption. Body composition analysis showed a significant decrease in the non-fat carcass mass without a change in carcass fat or body water. This result suggests that depletion of lean body mass in mice bearing G361 melanoma arises from the production of PIF. © 1999 Cancer Research Campaign


*British Journal of Cancer* (1999) **80**(11), 1734–1737
© 1999 Cancer Research Campaign
Article no. bjoc.1999.0590

### Role of a proteolysis-inducing factor (PIF) in cachexia induced by a human melanoma (G361)

**PT Todorov, WN Field and MJ Tisdale**

Pharmaceutical Sciences Institute, Aston University, Birmingham B4 7ET, UK

**Summary** Human melanoma, G361, which induces cachexia in nude mice, has been shown to produce a proteolysis-inducing factor (PIF) of $M_r$ 24 000, which is immunologically identical to that isolated from a cachexia-inducing murine tumour (MAC16). Biosynthetic labelling of G361 cells using a combination of [$^{35}$S]sulphate and [6-$^3$H]glucosamine gave a single component of $M_r$ 24 000 after affinity chromatography employing a murine monoclonal antibody. The material contained both radiolabels and, after digestion with peptide *N*-glycosidase F, two fragments were produced of $M_r$ 14 000 and 10 000 also containing both radiolabels. Digestion with *O*-glycosidase produced three fragments of $M_r$ 14 000, 6000 and 4000, the first two of which contained both radiolabels, while the third only contained $^3$H. This digestion pattern is the same as that previously observed with PIF from the MAC16 tumour and is commensurate with one N-linked sulphated oligosaccharide chain of $M_r$ 10 000, one O-linked sulphated oligosaccharide chain of $M_r$ 6000 and a central polypeptide chain of $M_r$ 4000 with some residual carbohydrate. When PIF from G361 cells was administered to female NMRI mice (20 g) a pronounced depression of body weight (1.36 ± 0.36 g; $P < 0.0001$ from control) was observed over a 24 h period without a decrease in either food or water consumption. Body composition analysis showed a significant decrease in the non-fat carcass mass without a change in carcass fat or body water. This result suggests that depletion of lean body mass in mice bearing G361 melanoma arises from the production of PIF.

**Keywords:** cancer cachexia; proteolysis-inducing factor; G361 melanoma; sulphated glycoprotein

Cachexia is the most common paraneoplastic syndrome in cancer with about one-half of untreated cancer patients having lost some weight at the time of presentation (De Wys et al, 1980). There is a reduction in both body fat and lean body mass, but while there is marked depletion of skeletal muscle, the non-muscle protein compartment is relatively preserved, thus differentiating cancer cachexia from simple starvation (Fearon, 1992).

A number of cytokines, including tumour necrosis factor-α (TNF-α) (Beutler and Cerami, 1986), interleukin-1 (IL-1) (Moldawer et al, 1988), interleukin-6 (IL-6) (Strassman et al, 1992), interferon-γ (IFN-γ) (Matthys et al, 1991) and leukaemia inhibitory factor (LIF) (Mori et al, 1989) have been suggested to play a role in the cachectic process. However, although cytokine production has been associated with the development of the cachectic syndrome induced by some human tumour cell lines, in others cachexia can occur in the absence of cytokine production, suggesting that other factors are also involved (Kajimura et al, 1996). Studies from our own laboratory have provided evidence for the production of a proteolysis-inducing factor (PIF) by both murine and human tumours (Todorov et al, 1996*a*). This factor produces loss of skeletal muscle by decreasing protein synthesis and increasing protein degradation (Lorite et al, 1997). Unlike the cytokines, PIF is capable of inducing protein degradation in isolated skeletal muscle (Todorov et al, 1996*b*) and produces a state of cachexia in non-tumour-bearing mice without a reduction in food and water intake.

*Received 27 October 1998*
*Revised 1 March 1999*
*Accepted 2 March 1999*

*Correspondence to:* MJ Tisdale

Even in cytokine-secreting tumours other factors may be involved in the development of cachexia, particularly in the erosion of skeletal muscle mass. In the present study, the production of PIF has been determined in a human melanoma cell line, G361. This tumour has been reported to induce severe cachexia in tumour-bearing nude mice and this has been correlated with the expression of LIF (Mori et al, 1991). In addition, biosynthetic labelling studies have been performed to determine if human PIF contains the same number and size of oligosaccharide chains as previously reported in the mouse (Todorov et al, 1997) as well as the same linking to the polypeptide core.

### MATERIALS AND METHODS

### Materials

[D-6-$^3$H]Glucosamine hydrochlorinol (sp. act. 18.5 Ci mmol$^{-1}$) and sodium [$^{35}$S] sulphate (sp. act. 10–100 mCi mmol$^{-1}$) were purchased from Amersham International (Bucks, UK). Tissue culture medium and fetal bovine serum (FBS) were from Life Technologies Inc (Paisley, UK). Peptide *N*-glycosidase F (PNGase F) and endo-α-*N*-acetylgalactosaminidase (*O*-glycosidase) were obtained from Oxford Glycosystems Ltd (Oxford, UK). Pure strain NMRI mice were obtained from our own inbred colony and were fed a rat and mouse breeding diet (Special Diet Services, Wiltham, UK). Monoclonal antibody was concentrated from the tissue culture supernatant of a hybridoma using a protein A-Sepharose column as described (Todorov et al, 1996*b*). The human melanoma cell line, G361, was obtained from the European Collection of Cell Cultures (Porton Down, Wilts, UK). Affi-Gel Hz was purchased from Bio-Rad (Hemel Hempstead, UK).

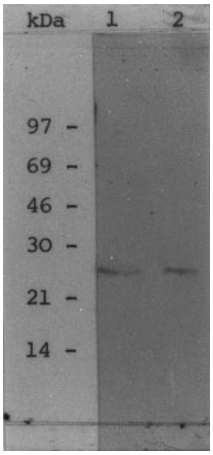

**Figure 1** Immunoblot of PIF affinity purified from G361 (lane 1) and MAC16 (lane 2) cells detected using monoclonal antibody, which had been biotinylated using the ECL protein biotinylation module (Amersham UK). Samples were electrophoresed on 15% sodium dodecyl sulphate polyacrylamide gels, transferred to nitrocellulose membranes blocked with 5% Marvel in 0.15% Tween-20 in PBS. Filters were washed and incubated for 1 h with 10 μg ml$^{-1}$ biotinylated antibody. After further washing, filters were incubated for a further 1 h with streptavidin–horseradish peroxidase conjugate (Amersham) and bands were detected with an emission chemiluminescence (ECL) system

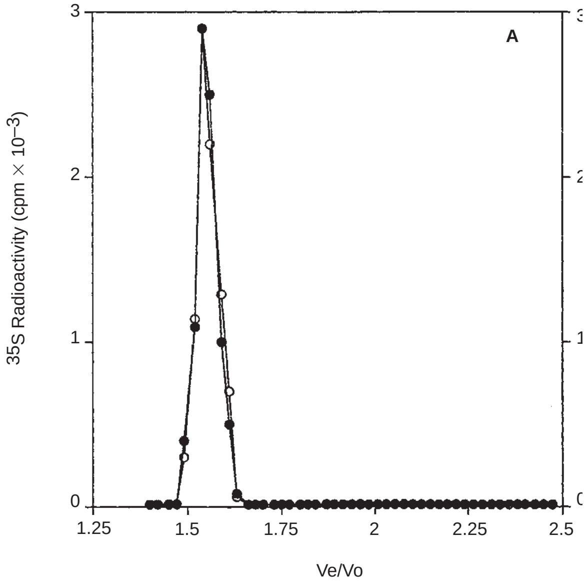

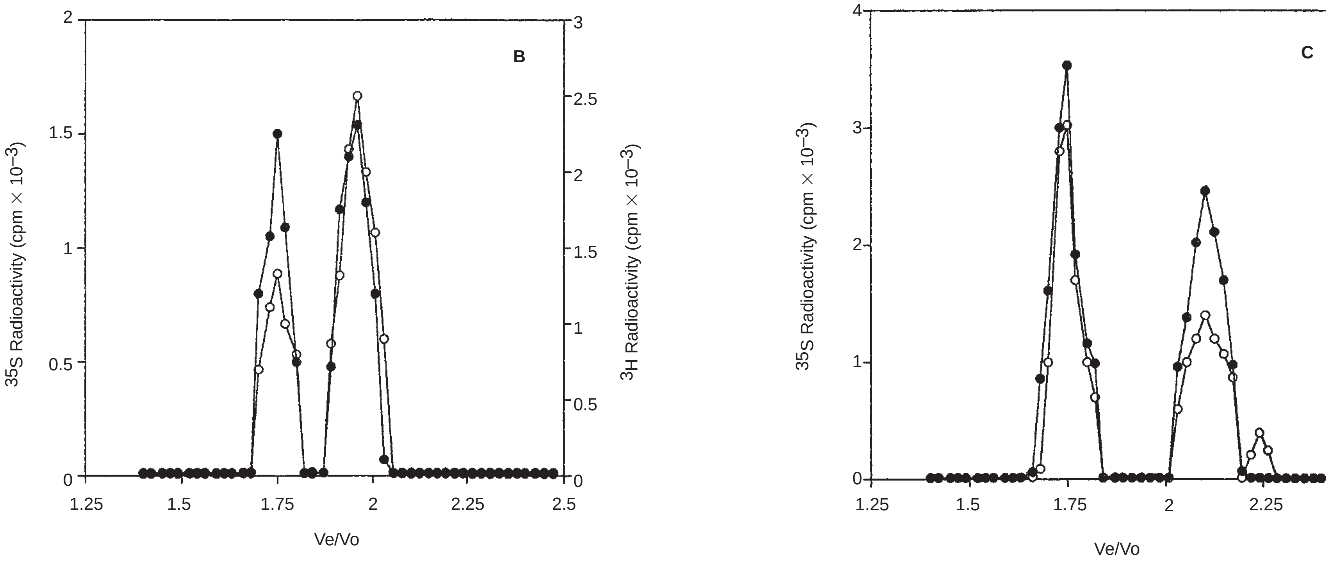

**Figure 2** Elution profile of biosynthetically labelled, affinity purified PIF isolated from G361 cells and chromatographed on a Sephadex G-50 column (40 × 1.5 cm). The column was equilibrated with 10 mM Tris–HCl, pH 7.0 containing 0.2 M sodium chloride and eluted at 6 ml h$^{-1}$. Fractions (0.7 ml) were collected and assayed for $^{35}$S (●) and $^{3}$H (○) radioactivity using a dual counting procedure. (**A**) Without treatment. (**B**) After incubation for 24 h with protease-free recombinant PNGase F (1 unit per 20 μl) at pH 8.0 and 37°C. The enzyme was also free of endo-α-*N*-acetylgalactosaminidase H/endo-α-*N*-acetylgalactosaminidase F and glycosidase. (**C**), after incubation for 20 h with *O*-glycosidase (1 m unit per 20 μl) in 100 mM phosphate–citrate, pH 6.0. The enzyme was free of protease and contaminating glycosidases. Treatment with the incubation buffers alone had no effect on the elution position of the $M_r$ 24 000 material

### Cell culture and radiolabelling procedures

G361 cells were maintained in McCoys 5a medium supplemented with 2 mM glutamine and 10% FBS under an atmosphere of 5% carbon dioxide ($CO_2$) in air. Cells were plated in McCoys 5a medium containing $Na_2{}^{35}SO_4$ (1 μCi ml$^{-1}$) and [$^3$H]glucosamine (2 μCi ml$^{-1}$) and allowed to grow for 48 h at 37°C under an atmosphere of 5% $CO_2$ in air. Cells were removed from the substratum with trypsin–EDTA and sedimented by low speed centrifugation (1500 rpm for 5 min on a bench-top centrifuge) followed by washing in phosphate-buffered saline (PBS). The cell pellet was resuspended in 1 ml of 10 mM Tris–HCl (hydrochloric acid), pH 8.0, containing 0.5 mM phenylmethylsulphonyl fluoride (PMSF), 0.5 mM EGTA and 1 mM dithiothreitol, and dissociated using an ultrasonic oscillator. Debris was removed by centrifugation (15 000 rpm for 20 min); solid ammonium sulphate (80% w/v) was added to the supernatant and the mixture was stored overnight at 4°C. The precipitated proteins were collected by

© 1999 Cancer Research Campaign

**Table 1** Effect of PIF isolated from G361 cells on body weight and body composition of female NMRI mice[a]

| Parameter | Control | PIF | $P$ [b] |
|---|---|---|---|
| Weight change over 24 h (g) | + 1.02 ± 0.39 | –1.36 ± 0.36 | 0.0001 |
| Food intake (g) | 3.6 | 3.3 | NS |
| Water intake (ml) | 3.8 | 3.8 | NS |
| Body water (%) | 67.2 ± 0.3 | 66.4 ± 1.5 | NS |
| Non-fat (g) | 7.26 ± 0.08 | 6.19 ± 0.32 | 0.01 |
| Fat (g) | 1.39 ± 0.07 | 1.44 ± 0.13 | NS |

[a]Values are given as mean ± s.e.m. for five mice per group. [b]Differences between control and PIF groups were determined by Student's *t*-test.

centrifugation and the pellet resuspended in the sonicating buffer. Salt was removed by ultrafiltration with an Amicon filtration cell containing a membrane filter with a molecular weight cut-off of 10 000 against the same solution. The concentrated sample was loaded onto an affinity column containing mouse monoclonal antibody coupled to Affi-Gel Hz (Todorov et al, 1996*b*) equilibrated with 10 mM Tris–HCl, pH 8.0 at 4°C. After overnight circulation at a flow rate of 5 ml $h^{-1}$ the column was washed with 10 mM Tris–HCl, pH 8.0, and the retained material was eluted with 100 mM glycine HCl, pH 2.5. The fractions were counted for radioactivity using a Packard 2000 CA liquid scintillation analyser and the fractions containing radioactivity were neutralized with 1 M Tris–HCl, pH 8.0, concentrated by Amicon filtration and subjected to exclusion chromatography on a Sephadex G-50 column. Non-radioactive PIF was also purified from MAC16 and G361 cells using affinity chromatography.

### Animal studies

Female NMRI mice (five per group; average weight 20.6 g) were injected intravenously (i.v.) into the tail vein with PIF (7 μg protein in 100 μl PBS) at 1.5 h intervals over a 6 h period (total of four injections). Control animals received PBS alone. Body weight and food and water intake were monitored and the animals were sacrificed 24 h after the first injection. Each carcass was weighed, placed in an oven at 80°C until constant weight was reached and re-weighed. The total fat content of the carcass was determined by the method of Lundholm et al (1980). The residue was the non-fat mass. The water content was calculated from the difference between the wet and dry weights.

### RESULTS

PIF was isolated from G361 cells by an initial 40% ammonium sulphate ($(NH_4)_2SO_4$) precipitation followed by affinity chromatography using monoclonal antibody specific to PIF (Todorov et al, 1996*b*). Western blotting of immunoaffinity purified material gave evidence for a single antigen of $M_r$ 24 000 identical to that isolated from the MAC 16 tumour (Figure 1).

In order to evaluate the number, size and attachment of the oligosaccharide chains to the central polypeptide core in human PIF, G361 cells were metabolically labelled with $^{35}SO_4$ and [6-$^3$H]-glucosamine, which specifically labels the carbohydrate fraction. Immunoaffinity purification gave a single band of radioactivity containing both radioisotopes. The apparent molecular weight as determined by exclusion chromatography on Sephadex G-50 was 24 000 in comparison with standard proteins (Figure 2A). Re-chromatography of this material after overnight digestion with PNGase F produced two bands of radioactivity eluting at positions corresponding to $M_r$ 14 000 and 10 000 (Figure 2B). Both fragments contained $^3$H and $^{35}$S. Digestion of the $M_r$ 24 000 material with *O*-glycosidase followed by re-fractionation on Sephadex G50 showed conversion to three bands of radioactivity corresponding to $M_r$ 14 000, 6000 and 4000 (Figure 2C). The first two fragments contained both radiolabels, while the third only contained $^3$H.

To evaluate the biological effectiveness of PIF produced by G361, affinity-purified material was administered i.v. to female NMRI mice and changes in body weight and body composition were determined after 24 h (Table 1). Animals receiving PIF showed a 6.6% decrease in body weight over 24 h, while PBS-treated controls showed a 5% increase in body weight. The difference was highly significant (Table 1). There was no difference in food and water consumption between the groups. Body composition analysis showed a significant reduction in the non-fat carcass mass (Table 1), with no change in water or fat content. This suggests that PIF produced by the G361 cell line is biologically active.

### DISCUSSION

Using a monoclonal antibody derived from splenocytes of mice bearing the cachexia-inducing MAC16 tumour we have been able to identify an antigen of $M_r$ 24 000, which is immunologically identical both in the MAC16 tumour and in the urine of patients with cachexia and a variety of tumour types (Todorov et al, 1996*a*). This antigen is a sulphated glycoprotein (Todorov et al, 1997) with a central polypeptide core, the amino acid sequence of which is also identical in both mouse (Todorov et al, 1996*a*) and man (Cariuk et al, 1997). Material from both sources caused a rapid loss of body weight in non-tumour-bearing mice by specific depletion of the non-fat carcass mass (Todorov et al, 1996*a*; Cariuk et al, 1997).

In the present study material of identical molecular weight to that isolated from the MAC16 tumour has been purified from human melanoma cells, G361, confirming the tumour origin of the human product. This material has also identical immunoreactivity with the mouse monoclonal antibody, which recognizes the carbohydrate chains in the glycoprotein (Todorov et al, 1997). In addition, the number, size and attachment of the oligosaccharide chains to the peptide core is the same in the G361 product as that isolated from the MAC16 tumour. Incubation of PIF from G361 cells with recombinant protease-free PNGase F, which specifically cleaves the *N*-acetylglucosamine–asparagine bond of N-linked oligosaccharides (Tarentino et al, 1985), yielded two fragments of $M_r$ 14 000 and 10 000, both of which were sulphated, as previously observed with PIF isolated from the MAC16 tumour (Todorov et al, 1997). The fragment of $M_r$ 10 000 has been attributed to a N-linked oligosaccharide chain, while the fragment of $M_r$ 14 000 represents the peptide core and residual oligosaccharide. Treatment of PIF from G361 cells with *O*-glycosidase, which has stringent specificity for the core structure Gal β1→3 Gal NAc α1-Ser/Thr, yielded a fragment of $M_r$ 6000, representing an O-linked sulphoglycan chain (Todorov et al, 1997) and a fragment of $M_r$ 4000 labelled with $^3$H alone, representing the core peptide with some carbohydrate attached. Thus the size and attachment of the oligosaccharide chains in the human PIF is the same as previously reported in the mouse (Todorov et al, 1997).

© 1999 Cancer Research Campaign

Production of cachexia by G361 cells in nude mice has previously been attributed to the expression of LIF (Mori et al, 1991). LIF has been suggested to play a role in the cancer cachexia syndrome through the ability to decrease lipoprotein lipase (LPL) activity (Marshall et al, 1994). Inhibition of LPL would prevent adipocytes extracting fatty acids from plasma lipoproteins for storage, resulting in a net flux of lipid into the circulation. No mechanism has been proposed for catabolism of skeletal muscle by LIF. Inhibition of LPL alone would not produce cachexia, since none is seen in type 1 hyperlipidaemia caused by an inherited deficiency of LPL. Although exogenously administered, recombinant LIF has been shown to induce loss of body weight in experimental animals, this has been reported as only occurring at dose levels near the toxic limit (Metcalf et al, 1990). The loss of body weight was ascribable to a complete loss of subcutaneous and intra-abdominal fat with no change in liver and kidney weights, suggesting that the weight loss was not a true cachexia. This suggests that factors other than LIF may be responsible for the cachexia induced by G361 cells.

In this study we have shown that PIF, purified from G361 cells, is capable of producing loss of body weight in mice, with specific loss of the carcass non-fat mass. There was no loss of adipose tissue and no decrease in food intake as would be expected with LIF. Thus it is likely that G361 melanoma cells produce cachexia as a result of expression of PIF rather than LIF.

This study has shown that human melanoma cells produce a PIF which is structurally and functionally identical with the mouse factor. Further studies are required to establish the degree of homology of the gene coding for the central polypeptide chain in the two species.

### ACKNOWLEDGEMENT

This work has been supported by Pfizer Inc.

### REFERENCES

Beutler B and Cerami A (1986) Cachectin and tumour necrosis factor as two sides of the same biological coin. *Nature* **320**: 584–588

Cariuk P, Lorite MJ, Todorov PT, Field WN, Wigmore SJ and Tisdale MJ (1997) Induction of cachexia in mice by a product isolated from the urine of cachectic cancer patients. *Br J Cancer* **76**: 606–613

De Wys WD, Begg C, Lavin PT, Band PR, Bennett JM, Bertino JR, Cohen MH, Douglass HD, Engstrom PF, Ezdinlie Z, Horton J, Johnson GJ, Moertel CG, Oken MM, Perla C, Rosenbaum C, Sinerstein MN, Skeel RT, Sponzo RW and Tormey DC (1980) Prognostic effect of weight loss prior to chemotherapy in cancer patients. *Am J Med* **69**: 491–497

Fearon KC (1992) The mechanisms and treatment of weight loss in cancer. *Proc Nutr Soc* **51**: 251–265

Kajimura N, Iseki H, Tanaka R, Ohue C, Otsubo K, Gyoutoku M, Sasaki K, Akiyama Y and Yamaguchi K (1996) Toxohormones responsible for cancer cachexia syndrome in nude mice bearing human cancer cell lines. *Cancer Chemother Pharmacol* **38**: S48–S52

Lorite MJ, Cariuk P and Tisdale MJ (1997) Induction of muscle protein degradation by a tumour factor. *Br J Cancer* **76**: 1035–1040

Lundholm K, Edström S, Karlberg I, Ekman L and Schersten T (1980) Relationship of food intake, body composition and tumour growth to host metabolism in non-growing mice with sarcoma. *Cancer Res* **40**: 2515–2522

Marshall MK, Doerrler W, Feingold KR and Grunfeld C (1994) Leukemia inhibitory factor induces changes in lipid metabolism in cultured adipocytes. *Endocrinology* **135**: 141–147

Matthys P, Heremans H, Opdenakker G and Billau A (1991) Anti-interferon-γ antibody treatment, growth of Lewis lung tumours in mice and tumour-associated cachexia. *Eur J Cancer* **27**: 182–187

Metcalf D, Nicola NA and Gearing DP (1990) Effects of injected leukemia inhibitory factor on hematopoietic and other tissues in mice. *Blood* **76**: 50–56

Moldawer LL, Andersson C, Gelin J and Lundholm KG (1988) Regulation of food intake and hepatic protein synthesis by recombinant-derived cytokines. *Am J Physiol* **254**: G450–G456

Mori M, Yamaguchi K and Abe K (1989) Purification of lipoprotein lipase-inhibitory protein produced by a melanoma cell line associated with cancer cachexia. *Biochem Biophys Res Commun* **160**: 1085–1092

Mori M, Yamaguchi K, Honda S, Nagasaki K, Ueda M, Abe O and Abe K (1991) Cancer cachexia syndrome developed in nude mice bearing melanoma cells producing leukemia-inhibitory factor. *Cancer Res* **51**: 6656–6659

Smith KL and Tisdale MJ (1993) Mechanism of muscle protein degradation in cancer cachexia. *Br J Cancer* **68**: 314–318

Strassman G, Fong M, Kenney JS and Jacob CO (1992) Evidence for the involvement of interleukin 6 in experimental cancer cachexia. *J Clin Invest* **89**: 1681–1684

Tarentio AL, Gomez CM and Plummer TH Jr (1985) Deglycosylation of asparagine-linked glycans by peptide:N-glycosidase F. *Biochem* **24**: 4665–4671

Todorov P, Cariuk P, McDevitt T, Coles B, Fearon K and Tisdale M (1996*a*) Characterization of a cancer cachectic factor. *Nature* **379**: 739–742

Todorov PT, McDevitt TM, Cariuk P, Coles B, Deacon M and Tisdale MJ (1996*b*) Induction of muscle protein degradation and weight loss by a tumor product. *Cancer Res* **56**: 1256–1261

Todorov PT, Deacon M and Tisdale MJ (1997) Structural analysis of a tumor-produced sulfated glycoprotein capable of initiating muscle protein degradation. *J Biol Chem* **272**: 12279–12288

© 1999 Cancer Research Campaign 
